# Historical colonization and dispersal limitation supplement climate and topography in shaping species richness of African lizards (Reptilia: Agaminae)

**DOI:** 10.1038/srep34014

**Published:** 2016-09-27

**Authors:** W. Daniel Kissling, Anne Blach-Overgaard, Roelof E. Zwaan, Philipp Wagner

**Affiliations:** 1 Institute for Biodiversity and Ecosystem Dynamics (IBED), University of Amsterdam, P.O. Box 94248, 1090 GE, Amsterdam, The Netherlands; 2 Section for Ecoinformatics & Biodiversity, Department of Bioscience, Aarhus University, Ny Munkegade 114, DK-8000 Aarhus C, Denmark; 3 Zoologische Staatssammlung München, Münchhausenstr. 21, D81247 München, Germany; 4 Villanova University, Department of Biology, 800 Lancaster Avenue, Villanova, PA 19085, USA

## Abstract

To what extent deep-time dispersal limitation shapes present-day biodiversity at broad spatial scales remains elusive. Here, we compiled a continental dataset on the distributions of African lizard species in the reptile subfamily Agaminae (a relatively young, Neogene radiation of agamid lizards which ancestors colonized Africa from the Arabian peninsula) and tested to what extent historical colonization and dispersal limitation (i.e. accessibility from areas of geographic origin) can explain present-day species richness relative to current climate, topography, and climate change since the late Miocene (~10 mya), the Pliocene (~3 mya), and the Last Glacial Maximum (LGM, 0.021 mya). Spatial and non-spatial multi-predictor regression models revealed that time-limited dispersal via arid corridors is a key predictor to explain macro-scale patterns of species richness. In addition, current precipitation seasonality, current temperature of the warmest month, paleo-temperature changes since the LGM and late Miocene, and topographic relief emerged as important drivers. These results suggest that deep-time dispersal constraints — in addition to climate and mountain building — strongly shape current species richness of Africa’s arid-adapted taxa. Such historical dispersal limitation might indicate that natural movement rates of species are too slow to respond to rates of ongoing and projected future climate and land use change.

What determines the distribution of life on Earth is a fundamental question in ecology, evolution, conservation, and global change biology^1^. Over the last decades, current climate and environmental heterogeneity have been tested widely as drivers of broad-scale patterns of species richness^2^,^3^. More recently, deep-time historical factors such as paleoclimatic changes^4^,^5^,^6^,^7^ or mountain building processes^8^ have been investigated, suggesting that they might also play an important role in shaping macro-scale species richness. In addition, though less acknowledged, a set of historical drivers has been related to dispersal constraints, i.e. reflecting dispersal limitation due to physical barriers, time-limited expansion from refugia, or the geography of historical colonization. For instance, geographic accessibility from glacial refuges (i.e. post-glacial dispersal limitation) can explain a large part of the spatial variation in European tree diversity^9^. Similarly, the diversity of widespread and endemic mammals across Europe can be partly explained by dispersal constraints related to an east–west colonization from Asia^10^. However, beyond Europe the importance of these dispersal constraints for broad-scale patterns of species richness has been little explored. Fortunately, newly available software packages for generating maps of accessibility from areas of origin now facilitate the testing of how dispersal constraints might influence macro-scale patterns of species distributions and biodiversity^11^.

The African continent harbours a wide variety of ecosystems and climates, including deserts, steppes, grasslands, shrublands, woodlands, savannahs, and dry and wet forests^12^,^13^. Although Africa had been largely covered by tropical rainforests in the early Eocene *c.* 55 mya^5^,^14^,^15^, the continent today is mostly covered by arid or semi-arid landscapes such as deserts, open grasslands, or shrublands^13^. Compared to other tropical regions such as South America or Southeast Asia, Africa’s species richness is much lower, a reason why it has been referred to as ‘the odd man out’^16^. This lower diversity is possibly caused by late Cenozoic climate cooling and aridification^15^, which led to severe losses of tropical rainforests^5^ and the expansion of open habitats and arid-adapted vegetation^14^,^17^. In addition, the formation of the Sahara desert, estimated to be around 7 mya^18^ (but see ref. 19), caused a major dispersal barrier for many species^20^, and in combination with paleoclimatic changes and topography might have triggered radiations of arid-adapted taxa^21^,^22^,^23^. Moreover, the disjunct distributions of numerous African plants and animals suggest that arid corridors in East Africa, the Sahel, and North Africa have had a major influence on the distributions and historical dispersal routes of many taxa^24^,^25^,^26^. However, to what extent arid corridors, geographic colonization and historical dispersal routes have shaped present-day species richness across Africa remains little explored.

With ~10,000 species worldwide, reptiles are a diverse group of vertebrates. In biogeographic analyses, reptiles are often under-represented compared to other terrestrial vertebrates^27^ because species-level global distributions are largely unmapped for most parts of the world (www.gardinitiative.org). The squamates (scaled reptiles) are the largest living order of reptiles, comprising all lizards and snakes. Within the squamates, lizards in the family Agamidae are a morphologically and ecologically diverse Old World family occurring in Africa, Europe, Asia (including India) and Australia^26^. In Africa, the agamid lizards are among the most diverse and widespread squamates, with some species showing an extravagant breeding coloration in males. The African agamid lizards exclusively occur in arid habitats such as savannahs, making them an ideal group for biogeographic and evolutionary studies of Africa’s arid-adapted taxa^21^,^26^,^28^. The Agamidae in Africa probably have an East Asian origin, as supported by fossil evidence, molecular phylogenies, high species diversity in Southeast Asia, and a general absence in Gondwanan areas such as Madagascar and South America^26^,^28^. The ancestors of African agamids most likely colonized Africa through dispersal from Asia via the Arabian Peninsula^29^, probably through two hypothesized colonization routes^26^: one from East to West Africa (the Sahel corridor), and one from the Horn of Africa to south-western Africa (i.e. the arid corridor in the East). The African radiation of the subfamily Agaminae (including the genera *Acanthocercus, Agama, Pseudotrapelus, Trapelus* and *Xenagama*) is probably the youngest radiation^30^, with diversification beginning approximately 23 mya^28^. Subsequent radiations have taken place in Southern, East, West, and Northern Africa during the Miocene^28^. Diversification has probably been influenced by topographic heterogeneity^28^ and changes in paleoclimate^21^, but to what extent these factors can explain current species richness relative to other factors such as historical dispersal limitation and current climate remains unknown.

Here, we test to what extent continental colonization and historical dispersal routes can explain present-day species richness of agamid lizards (subfamily Agaminae) in Africa relative to current climate, topography, and paloeclimatic changes. We first compiled a comprehensive database of species occurrence records across the continent, then used species distributions models (SDMs) combined with expert-based knowledge to derive continental species distribution maps of agamid lizards, and finally aggregated all distributional information at *c.* 110 × 110 km resolution to quantify species richness across Africa (Fig. 1). In a second step, we simulated potential dispersal routes for the entire subfamily as spatial spread patterns from the Arabian Peninsula into the African continent, and then correlated this simulated accessibility with agamid species richness across Africa. Finally, we used the simulated dispersal spread patterns together with other predictor variables (incl. current climate, paleoclimate and topographic heterogeneity; Table 1) to test whether historical dispersal routes are a strong explanatory variable for current species richness after accounting for other factors. We used multi-predictor models, incl. both non-spatial and spatial regressions, to evaluate the relative importance with standardized coefficients and partial residual plots. We show that geographic colonization from the Arabian Peninsula and dispersal limitation is a key predictor of present-day species richness of agamid lizards, suggesting that deep-time dispersal constraints shape biodiversity across Africa.

## Results

### Species distributions and diversity

**Figure 1:**
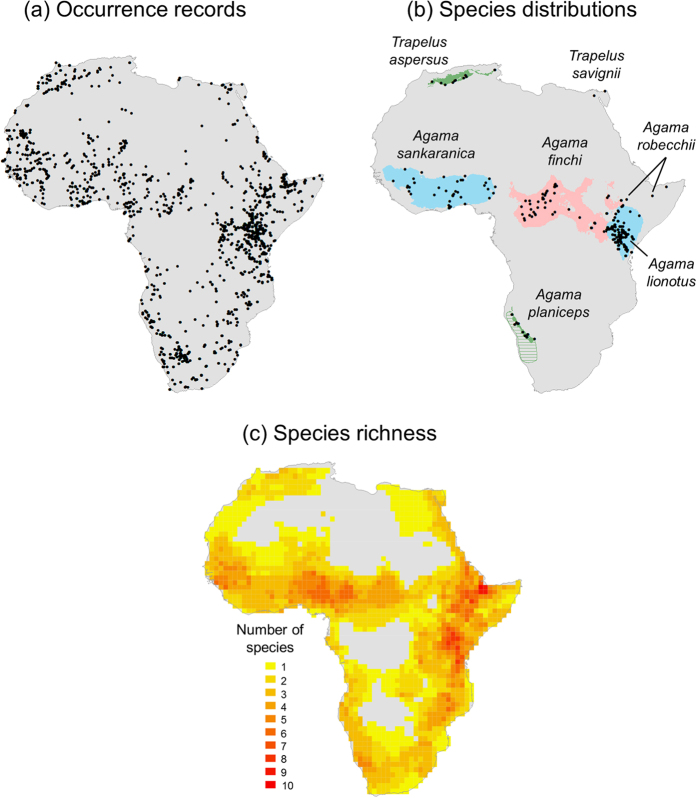
Distributional knowledge of agamid lizards across Africa. In (**a**), 1,454 geo-referenced and quality-checked records across all 74 species of agamid lizards are shown. The records are spatially unique at 10 × 10 km resolution. In (**b**), examples of binary species distribution maps at 10 × 10 km resolution are illustrated as derived from occurrence records and species distribution modelling. Species with <5 records (e.g. *Trapelus savignii* and *Agama robecchii*) were not modelled. Species with sample sizes 20 > x ≥ 5 (e.g. *Trapelus aspersus* and *Agama planiceps*) were modelled with a bioclimatic envelop (surface range envelope) model. Species with ≥20 records were modelled either with a bioclimatic envelop model (e.g. *Agama sankaranica*), machine-learning methods such as Maxent (e.g. *Agama finchi*), or generalized boosting models (e.g. *Agama lionotus*). In cases where a shortage of locality records did not allow to accurately predict a species distributional range (e.g. *Agama planiceps*), model predictions were complemented with expert-based range maps (shown with green lines). In (**c**), agamid species richness is illustrated, derived from summing up all individual species distributions for a grid in cylindrical equal area projection with 110 × 110 km resolution (equivalent to c. 1° × 1° near the equator). Species distributions were modelled using the statistical programming language R and maps were created using ArcGIS (version 10.2, ESRI, Redlands, CA, USA).

A total of 1,454 occurrence records were used to estimate the continental distributions of 74 agamid lizards across Africa (Fig. 1a). Species distributions varied from narrowly distributed species such as *Agama robecchii* and *Trapelus savignii* to widespread species such as *Agama sankaranica* and *Agama finchi* (Fig. 1b). The species richness map aggregated at *c.* 1° resolution (~12,000 km^2^ at the equator) revealed that agamid species richness peaked in East Africa, especially in mountainous regions, and in the Sahel corridor towards the west (Fig. 1c). In contrast, most parts of the Sahara as well as the Congolian rainforest were not inhabited by agamid lizards, and areas in the north of Africa as well as in the far South tended to have low species richness (Fig. 1c).

### Historical colonization and dispersal

**Figure 2:**
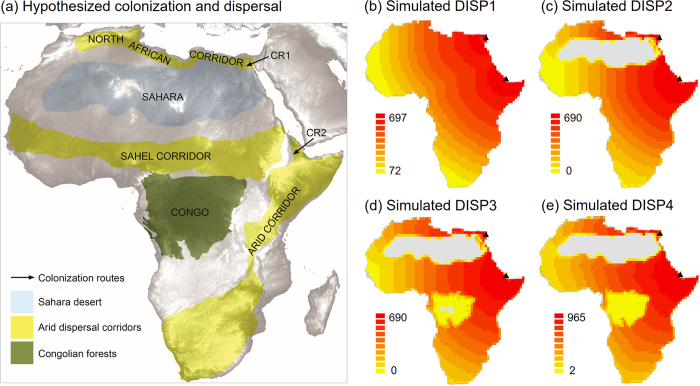
Hypothesized and simulated colonization and dispersal routes. (**a**) Hypothesized colonization routes of agamid taxa into Africa via the Sinai (CR1) or the Bab al-Mandab (CR2), the 27 km broad strait between the Arabian Peninsula and the African continent^26^. Subsequent dispersal of arid taxa into Africa has been hypothesised via arid dispersal corridors such as the North African corridor, the Sahel corridor, and the arid corridor from south-western Africa to the Horn of Africa^24^,^25^,^26^. Extents of the Sahara desert and Congolian forests were derived from Olson *et al*.^12^. (**b–e**) Simple dispersal spread patterns (DISP1–DISP4) simulated with KISSMig^11^, illustrating accessibility of the African continent from the origins CR1 and CR2 (black triangles). The scenarios represent DISP1 (no barriers, equally high suitability across Africa), DISP2 (Sahara desert unsuitable), DISP3 (like DISP2, but Congo forests with low suitability) and DISP4 (like DISP3, but arid corridors with high suitability and other cells with intermediate suitability). For further details of simulations see text and Table 1. Colonization was simulated using the statistical programming language R and maps were created using ArcGIS (version 10.2, ESRI, Redlands, CA, USA).

Phylogenetic, morphological and fossil data^28^,^29^,^31^,^32^ suggest that ancestors of the subfamily Agaminae have colonized Africa via the Arabian Peninsula (‘CR1’ and ‘CR2’ in Fig. 2a). Using a simple, raster-based, stochastic dispersal model^11^ we therefore simulated four generalized historical colonization patterns for the entire subfamily by generating maps of geographic accessibility from these areas of origin (Fig. 2b–e). The four dispersal scenarios (DISP1–4, summarized in Table 1) mainly differed in the way dispersal suitability of grid cells was quantified (for details see Table 1, Fig. 2b–e). All simulated dispersal scenarios (DISP1–DISP4) were significantly correlated with species richness of agamid lizards across Africa, but the correlation strengths differed among scenarios. Simulating dispersal into a homogeneous environment (DISP1, Table 1) was only weakly correlated with species richness (Spearman rank: *r* = −0.06, *p* < 0.002). When masking the Sahara desert as unsuitable (DISP2, Table 1), a stronger correlation between simulated dispersal and species richness emerged (*r* = 0.29, *p* < 0.001). Setting the Congo forests additionally with low suitability (DISP3, Table 1) produced an even higher correlation (*r* = 0.42, *p* < 0.001), and further assuming the highest suitability of colonization in arid corridors (DISP4, Table 1) produced the strongest correlation (*r* = 0.45, *p* < 0.001). The latter two dispersal scenarios (DISP3, DISP4) showed much higher correlations than most other tested environmental predictor variables (mean: *r* = 0.19; range: *r* = 0.03–0.43; *n* = 13). Moreover, a partitioning of the variation of species richness with respect to the four simulated dispersal scenarios showed that the variance explained by all four scenarios together (*R*^2^ = 0.23) could also be explained by only combining DISP1, DISP2 and DISP3 or DISP1, DISP2 and DISP4 (both with *R*^2^ = 0.23). Individually, DISP1 explained no variance in species richness (*R*^2^ = 0), DISP2 about 43% of the variance explained by all scenarios (*R*^2^ = 0.10), and DISP3 and DISP4 about 74% (*R*^2^ = 0.17). Hence, both DISP3 (with the Sahara desert masked and the Congo forest having low suitability) and DISP4 (additionally with higher suitability in arid corridors) were the most relevant dispersal scenarios to explain current species richness of agamid lizards.

### Relative importance of historical dispersal limitation

**Figure 3:**
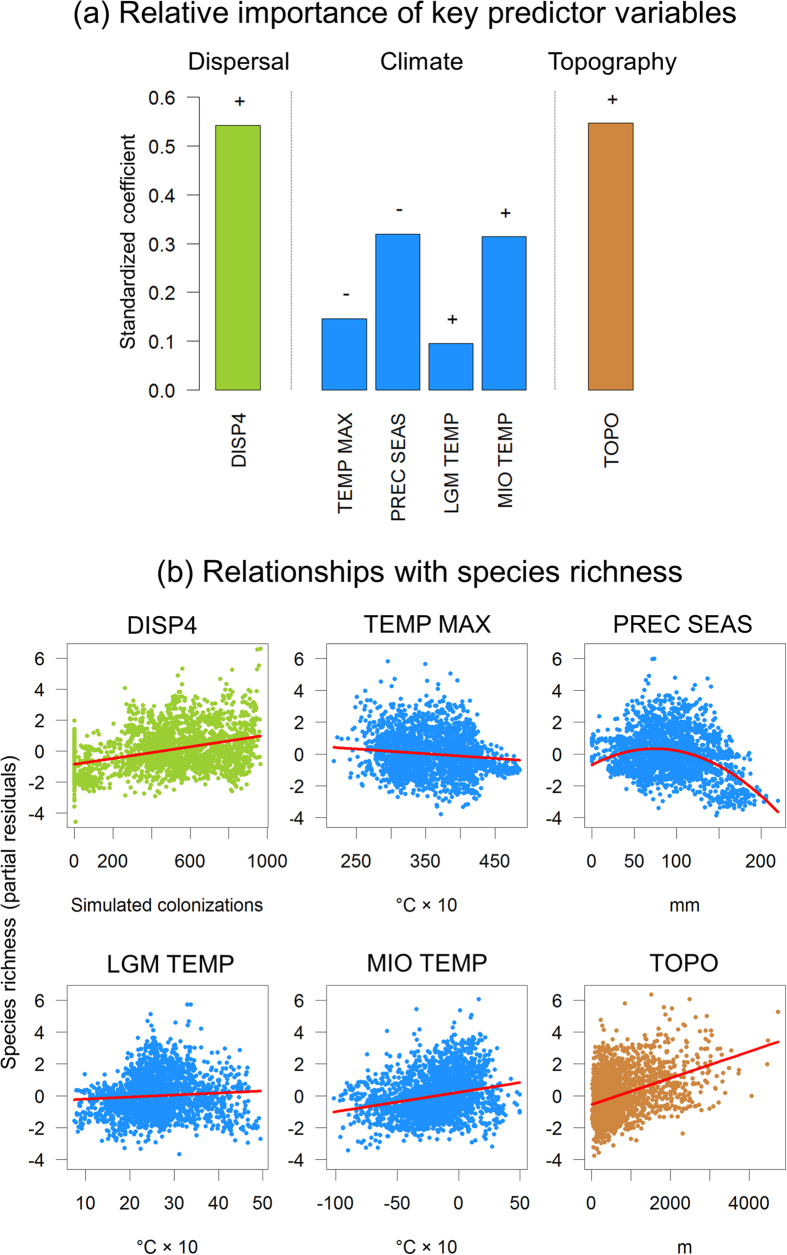
Effects of key predictor variables on species richness of agamid lizards across Africa. In (**a**), the relative importance (standardized coefficients) of six key predictor variables from the non-spatial regression model is illustrated. The variables are those that show significant effects in both spatial and non-spatial models (compare Table 2). The direction of effect is indicated as + or −. In (**b**), partial residual plots illustrate the relationship between a predictor and species richness once all other predictors have been statistically accounted for in a multiple-predictor model (see ‘OLS’ in Table 2). Abbreviations, units and sources of predictor variables are explained in Table 1. Each dot represents one 110 × 110 km grid cell. Plots were created using the statistical programming language R.

We used the standardized coefficients of non-spatial and spatial multi-predictor regression models to test the relative importance of dispersal scenarios and other predictor variables for explaining agamid species richness across Africa (Table 2 and Supplementary Material). A model selection with the Akaike Information Criterion (AIC)^33^ showed that a non-spatial ordinary least squares (OLS) regression model with twelve predictor variables (i.e. five variables related to current climate, five to past climate, one to topography, and one to historical dispersal limitation, i.e. DISP4) was the most parsimonious multivariate model (i.e. having the lowest AIC among all possible candidate models) (Table 2). This model explained about half of the continental variation in agamid species richness (*R*^2^ = 0.45). Because spatial autocorrelation was present in OLS model residuals (see Moran’s *I* and its p-value in Table 2) we also implemented a spatial simultaneous autoregressive (SAR) model^34^ with the same predictor variables. This allowed to account for residual spatial autocorrelation and captured most of the variation in species richness (*R*^2^_*FULL*_ = 0.89). In both OLS and SAR regressions, the simulated dispersal scenario (DISP4) was among the most important predictor variables (i.e. high standardized coefficients in Table 2 and Fig. 3a). In the OLS model, DISP4 showed the strongest effect together with topography whereas climate variables were much less important (Fig. 3a). In the SAR model, DISP4 was of similar importance to precipitation seasonality and LGM temperature anomaly (Table 2). Overall, the effect of DISP4 on species richness of agamid lizards was positive, indicating that areas with a high accessibility from the Arabian Peninsula tended to have more agamid species than areas with low accessibility further away (Fig. 3b). A multiple regression model including DISP3 instead of DISP4 yielded qualitatively similar results (see Supplementary Material), except that the effect of DISP3 was less pronounced than DISP4 (consistent with the Spearman rank correlations from the univariate analyses). Generally, the multi-variate analyses confirmed the hypothesis that historical dispersal limitation at a continental scale has left a strong imprint on present-day species richness of agamid lizards.

### Effects of current and past climate

Current and past climate also played a major role in explaining species richness of agamid lizards (Table 2). For present-day climate, the maximum temperature of the warmest month as well as precipitation seasonality were important predictors in both OLS and SAR models (Table 2). The effect of maximum temperature of the warmest month was negative (Fig. 3b), indicating that areas with temperature extremes (i.e. the Sahara desert) are colonized by very few agamid lizards. Precipitation seasonality showed a hump-shaped relationship with species richness (Fig. 3b), suggesting that regions with intermediate levels of precipitation seasonality tend to have higher species richness than areas with either low or high precipitation seasonality.

Two paleoclimatic predictor variables were consistently among the most important predictors in both OLS and SAR models (Table 2). Anomaly in annual mean temperature between the Last Glacial Maximum (*c.* 21,000 years ago) and the present had a positive effect on species richness (Fig. 3b), reflecting a high number of lizard species in areas which had been exposed to relatively strong Quaternary temperature oscillations. However, the magnitude of this effect (i.e. standardized coefficient) varied between OLS and SAR models (Table 2). Anomaly in annual mean temperature between the late Miocene (*c.* 11.61–7.25 mya) and the present also showed a positive relationship with species richness (Fig. 3b). This reflected that areas with warmer temperatures today than in the late Miocene tended to have more lizard species than areas which are relatively colder today than in the past. Other included climatic predictor variables also showed relationships with species richness, but the statistical significance of these effects was not consistent between OLS and SAR models (Table 2). We therefore consider these additional effects to be too uncertain for robust statistical and ecological inference.

### Topography

Topography played a key role in explaining lizard species richness in addition to historical dispersal limitation and current and past climate (Table 2, Fig. 3a). More species of agamid lizards were found in areas with high topographic heterogeneity compared to relatively flat areas (Fig. 3b). This suggests a strong role of mountains for the generation and maintenance of lizard species richness.

## Discussion

The effect of historical dispersal limitation on biodiversity is inherently difficult to test, and its importance in shaping species richness at macroscales thus remains elusive. We used a new software package^11^ to generate maps of accessibility from geographic areas of colonization via the Arabian Peninsula and then tested to what extent time-limited expansion and historical colonization of Africa could explain the continent-wide distribution of species richness of agamid lizards. We found that simulated spread patterns with low suitability in the Sahara desert and the Congo forests as well as high suitability in arid corridors were strong predictors of species richness, even when accounting for other predictor variables and spatial autocorrelation. In addition, current climate (i.e. precipitation seasonality and maximum temperature of the warmest month), paleoclimatic changes (i.e. late Miocene and LGM temperature anomaly), and topographic relief (i.e. elevational range) emerged as important drivers. This suggests that deep-time dispersal limitation — in addition to climate and topography — plays a key role in shaping species richness of Africa’s arid-adapted taxa.

The simulated dispersal scenarios assumed that ancestors of African Agaminae had colonization routes to Africa via the Arabian Peninsula (Fig. 2a). This is supported by phylogenetic and morphological data as well as fossil evidence. First, fossil evidence suggests an origin of the Agamidae as a whole family in East Asia^31^. Second, a time-calibrated phylogenetic tree of African agamid species shows that diversification in the subfamily Agaminae has mainly occurred over the last 10–20 myr^28^. In this phylogenetic tree, the genus *Agama* is the sister group to a clade that mainly occurs within the Horn of Africa (the African genera *Acanthocercus, Pseudotrapelus*, and *Xenagama*) or opposite of the Red Sea on the Arabian Peninsula (the two Arabian species of the genus *Acanthocercus*)^28^. Since the two Arabian species are basal to the African genera, a colonization via the Bab-al-Mandab (the 27 km broad strait between the Arabian Peninsula and the African continent) seems to be most likely. Third, the African species in the genus *Trapelus* are restricted to north of the Sahara, but the genus also ranges eastwards across Arabia to the western edge of India. Morphological and phylogenetic analyses indicate that *Trapelus* had more than one dispersal event into northern Africa^32^. Since the closest relative of most African *Trapelus* is found in Pakistan and India^29^, a dispersal route via the Sinai seems most plausible. This is consistent with the historical dispersal scenarios for some other North African lizard clades^35^. Hence, across African species in the subfamily Agaminae the available evidence suggests that their ancestors have colonized Africa from Asia via the Arabian Peninsula, including both the Bab al-Mandab and the Sinai^26^.

Using the two locations between the Arabian Peninsula and Africa as entry points for continental colonization, we simulated spatial spread patterns across Africa using four scenarios that differed in their suitability of grid cells for colonization and expansion (Table 1; Fig. 2). While such simulated accessibility maps from geographic origins are necessarily simplistic^11^, they do not require a priori knowledge about dispersal parameters and hence are a powerful tool for quantifying dispersal limitation when modelling macroscale patterns of species distributions and species richness^9^,^10^,^36^. Although the implemented dispersal scenarios did not account for environmental changes through time, they still represent major factors (i.e. barriers and corridors) that have been hypothesized to shape the dispersal ability of Africa’s taxa over millions of years. For instance, the Sahara desert has been suggested to be in place for at least 7 mya^18^. This timing is still debated^19^, but climate modelling experiments support a Late Miocene appearance of the Sahara desert^37^ and molecular divergence times of arid-adapted taxa also coincide with it (e.g. ref. 23). Hence, the Sahara has likely been a major dispersal barrier for low-mobility animals^22^ over millions of years, including agamid lizards^21^. Similarly, the Congo rainforests have been varying in extent over deep time^15^, but the general location of the core areas has been available for millions of years, e.g. in the Middle Pliocene^38^ and the Late Miocene^39^. For arid corridors, their specific timing of initiation and their ages are still debated, but an origin in the late Miocene is most likely as this relates to mountain uplift and associated rifting^40^ as well as major climatic changes in this epoch^14^,^18^. Overall, the simulated dispersal scenarios therefore represented major habitat features that likely have influenced the colonization and dispersal of agamid lizards (and other arid taxa) over millions of years.

Among the four simulations, the simulated accessibility with highest spread in arid corridors and lowest suitability in the Sahara desert and the Congo forests (DISP4) correlated best with observed patterns of present-day species richness. Dispersal scenario DISP3, which did not include a particularly high spread in arid corridors, showed slightly weaker support than DISP4. Overall, these results confirmed the habitat preferences of agamid lizards which occur predominantly in arid regions such as savannahs while they are absent in rainforests and dry and hot areas of the Saharan desert^26^. Arid corridors have been documented for many plant and animal groups in Africa^24^,^25^,^26^, but their importance for arid-adapted species might be strongly influenced by the presence and extent of the Sahara desert^18^ and the distribution and expansion of rainforest habitats through time^14^,^15^,^17^. This might explain the rather small differences between DISP3 and DISP4 in explaining species richness of agamid lizards. Overall, the high importance (i.e. large standardized coefficients) of these dispersal scenarios in multi-predictor models provide evidence that time-limited expansion (i.e. an east–west colonization from geographic areas of origin) in addition to current climate, paleoclimate and topography plays a key role to explain species richness of African lizards (Fig. 3).

**Figure 4:**
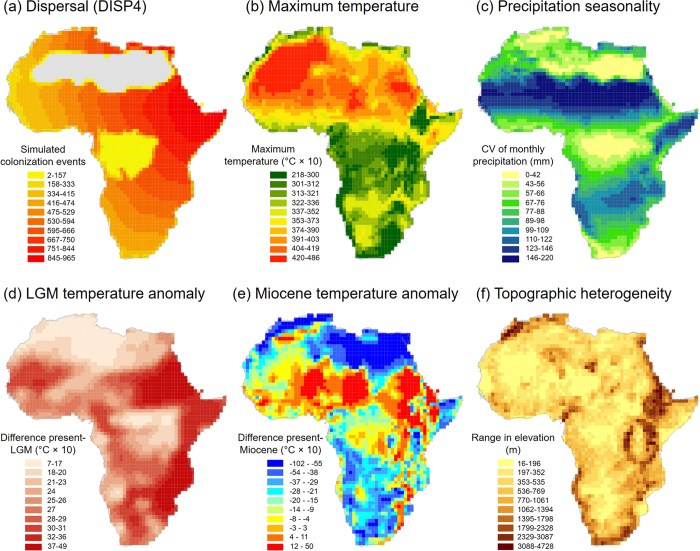
Geographic variation of key predictor variables. Variables include (**a**) the simulated accessibility via dispersal routes from colonization areas (compare ‘DISP4’ in Table 1), (**b**) present-day maximum temperature of the warmest month, (**c**) present-day precipitation seasonality (coefficient of variation of monthly precipitation values), (**d**) paleoclimatic changes (anomalies) in mean annual temperature between the Last Glacial Maximum (LGM) and the present, (**e**) paleoclimatic changes (anomalies) in mean annual temperature between the Miocene and the present, and (**f**) topographic heterogeneity (range in elevation). Maps are in WGS 1984 projection and show quantile classification. Created using ArcGIS (version 10.2, ESRI, Redlands, CA, USA).

In addition to historical dispersal limitation (Fig. 4a), current climate also played a key role for explaining species richness of agamid lizards. The general importance of temperature-related variables in our analysis is consistent with results from other broad-scale studies on species richness of lizards and reptiles in the Palearctic, North America, and Australia^2^. However, in contrast to Northern hemisphere studies we found a negative relationship between species richness and maximum temperature of the warmest month which indicates that the Saharan desert with its temperature extremes (red parts in Fig. 4b) is mostly uninhabitable for agamid lizards. This result corroborates the idea that physiological constraints related to temperature limit the distribution and diversity of ectotherms such as reptiles^41^. Ectotherm body temperatures closely follow environmental temperatures, and temperature extremes (both maximum and minimum) can therefore have a strong influence on the performance, metabolic rates, activity times, thermal limits and population dynamics of lizards^42^,^43^,^44^. Consequently, species richness of lizards and other ectotherms generally correlates more strongly with temperature than those of endotherms such as birds and mammals^2^,^41^. In addition to temperature, a hump-shaped relationship between species richness and current precipitation seasonality was revealed, suggesting that species richness peaks in areas of intermediate precipitation seasonality. In Africa, regions with intermediate precipitation seasonality (green areas in Fig. 4c) are mostly found along the Sahel corridor, in Northwest Africa, and in some parts of the arid corridor from the Horn of Africa to south-western Africa. In contrast, areas with the lowest precipitation seasonality (yellow in Fig. 4c) are located in the southern tips of Africa, in the tropical regions around the equator, and in parts of the Sahara and North Africa, whereas the highest precipitation seasonality (blue in Fig. 4c) is found in the southern parts of the Sahara north of the Sahel corridor. Recent analyses of desert-adapted vertebrate species suggest that future changes in precipitation (rather than temperature) might be a major threat for lizards in the Sahara-Sahel region^45^.

While it is widely accepted that current climate plays a major role in shaping ecological communities, paleoclimatic influences on species distributions and diversity often remain unexplored. Our results for arid-adapted lizards showed a positive relationship between LGM temperature anomaly and species richness. The direction of the effect contrasts with results on reptile diversity in Europe^4^ and is opposite to the effect of rainforest taxa such as palms for which high LGM temperature anomalies are usually associated with low species richness^46^. LGM temperature anomalies are roughly representative for the temperature oscillations of the whole Quaternary (the last 2.6 million years), as they cover almost the full Quaternary temperature range with a geographic pattern that is consistent with the orbitally driven climatic oscillations over at least a large portion of the period^47^. These repeated climatic cycles occurred on time-scales of 10–100 thousand years (Milankovitch oscillations) and have strongly shaped species distributions and diversity patterns at northern high latitudes^48^. However, their importance for tropical taxa still remains little explored. The positive relationship between agamid species richness and LGM temperature anomalies could indicate that arid-adapted lizards in tropical regions might have coped relatively well with the magnitude of such temperature oscillations, maybe because of their physiological tolerances and thermal adaptations to warm and dry environments. In Africa, areas of high LGM temperature anomaly are mostly found in the eastern part of Africa (dark red in Fig. 4d), where temperature differences of up to 5 °C have been inferred between the LGM and today. This temperature difference is relatively low when compared to northern high latitudes (>10 °C temperature anomaly)^47^,^48^, but it has still left a strong imprint on present-day species richness of African lizards.

Beyond Quaternary time scales, the positive relationship of species richness with Miocene temperature anomaly indicates that areas with higher temperatures today than in the late Miocene (*c.* 11.61–7.25 mya) coincide spatially with high lizard species richness. These areas are mostly located in East Africa and south of the Sahara (red in Fig. 4e). The Late Miocene (11.61–7.25 Ma) is generally considered to be a crucial period for the generation of arid regions in Africa^14^,^17^. Climate models and vegetation reconstructions suggest that in the late Miocene large parts of Africa had been covered by xerophytic shrublands, except the areas of the modern tropical rainforests and savannahs^39^. At this time, the extent of the Sahara was probably very small^18^,^39^. We hypothesize that the increasing aridification and the emergence of the Sahara in the late Miocene had a positive influence on agamid diversification, possibly via low extinction rates and increased speciation rates. This is supported by phylogenetic studies of North African lizards (genus *Agama*)^21^ and small mammals (genus *Elephantulus*)^20^ which suggest that late Miocene climate change has triggered allopatric speciation, especially in heterogeneous mountain regions.

Consistent with other taxa^3^, our analyses showed that high topographic heterogeneity coincides with high species richness. Mountains generally have steep climatic and habitat gradients in relatively small areas. This promotes spatial turnover of species adapted to different climatic and habitat conditions^49^ as well as opportunities for diversification through geographic isolation^50^. In Africa, areas with highly heterogeneous mountains mainly occur in East Africa, around Mount Cameroon, and along the coasts of southern and northwestern Africa (dark brown in Fig. 4f). Especially the East African mountains coincide with a high species richness of agamid lizards (Fig. 1c). The orogeny of these mountains is relatively young. Much of the currently high topography of the East African Rift system dates back to the Miocene^51^, and major mountain ranges such as the Rwenzori date only from the Pliocene^52^. We suggest that the relatively young mountain building in East Africa has influenced the Neogene diversification of agamid lizards, possibly by favouring genetic divergence and the splitting of lineages via allopatric speciation. A similar mechanism might apply to the lizards in the genus *Agama* in North Africa, where mountain building in combination with paleoclimatic changes have been invoked as a motor for diversification^21^. In the Sahara-Sahel region, there is also increasing evidence that local biodiversity hotspots and cryptic diversity are associated with small-sized water features within mountains^22^.

Our analyses employed a new approach for quantifying the extent at which deep-time dispersal limitation shapes present-day biodiversity at a continental scale. The simulation of deep-time dispersal spread patterns, though necessarily simplistic, emerges as a promising possibility to gain deeper insights into the importance of historical colonization and geographic accessibility for the distribution of species and species richness^9^,^11^,^36^. An alternative approach for quantifying historical migration is the reconstruction of ancestral areas within a phylogenetic framework^53^,^54^. This allows the quantification of timing of geographical colonization for specific clades^54^, but it requires a time-calibrated phylogeny and it does not allow to test simultaneously the relative effect of dispersal limitation and other factors on species richness in a spatially-explicit way. We therefore suggest that simulations of spatial spread patterns have great potential in macroecological analyses for generating and testing hypotheses about time-limited dispersal.

### Conclusions

Our study shows that deep-time dispersal limitation — together with climate and mountain topography — emerges as a key factor to explain present-day species richness of Africa’s arid-adapted taxa. To date, such simulations of historical spread patterns have mostly been used to assess time-limited dispersal from Pleistocene refugias of European trees^9^,^36^. We suggest that new applications to other regions, taxa, and deeper time periods will allow exciting new insights into how dispersal limitation shapes the broad-scale distribution of life on Earth. Future developments in modelling suitability maps over time might further improve the accuracy and realism of simulated spatial spread patterns. In the absence of direct dispersal estimates, such assessments of historical dispersal limitation can also provide first insights into how species and ecosystems might respond to future global change. For instance, strong historical dispersal limitation will make rapid movements and range dynamics in response to ongoing and future climate change unlikely, except if human-mediated dispersal of species causes major breakdowns of movement barriers^55^,^56^. More empirical and theoretical studies on dispersal limitation and human-assisted colonization and establishment are therefore urgently needed to better predict biodiversity responses to global change.

## Methods

### Species distributions and diversity

We collected a total of 16,802 occurrences records for nearly all African species in the reptile subfamily Agaminae (including genera *Acanthocercus, Agama, Pseudotrapelus, Trapelus* and *Xenagama*). Records came from a wide variety of sources, including the Global Biodiversity Information Facility (GBIF, http://data.gbif.org), HerpNET (http://www.herpnet.org/), additional museum collections, complementary literature, and private databases as well as field observations (sources summarized in Supplementary Material). We performed a careful quality check and meticulously scrutinized all records for any geographic or taxonomic issues. Records without latitude-longitude coordinates, doubtful records, geographic duplicates, and records from introduced species or those pre-dating 1950 were removed. We also rectified wrong species identifications and applied a number of taxonomic changes and corrections at subspecies, species and genera level to harmonize the taxonomy of the different datasets (for details see Supplementary Material). A total of 1,454 unique, geo-referenced and quality-checked occurrence records (i.e. 1–105 records per species; mean ± SD: 20 ± 20; median: 13) were finally retained (Fig. 1a). With this dataset we aim to contribute to ongoing efforts to compile essential biodiversity knowledge on species distributions^57^, specifically to the global assessment of reptile distributions (www.gardinitiative.org).

We used the 1,454 unique and geo-referenced records to estimate the geographic distributions of the 74 agamid lizard species at a 10 × 10 km resolution across Africa (for details see Supplementary Material). Since sample sizes (i.e. the number of unique occurrence records at 10 × 10 km resolution) differed among species, three different approaches to estimate continental species distributions were applied (Fig. 1b). For species with <5 records (*n* = 17), we used the observed records only, because sample size was too small for implementing SDMs. For species with ≥5 records, we implemented SDMs using the R package ‘biomod2’ version 2.1.9^58^, using two different strategies. For species with sample sizes 20 > *x* ≥ 5 (*n* = 31), a simple bioclimatic envelop SDM known as surface range envelope (SRE) was used^58^. For species with ≥20 records (*n* = 26), SRE as well as advanced SDMs based on machine-learning methods such as Maxent (MAX) and generalized boosting models (GBM) were implemented^58^. Model performance was assessed using the receiver operating characteristic curve (AUC)^59^ and the True Skill Statistic (TSS)^60^. All final SDMs showed good model performance (AUC: mean ± SD = 0.905 ± 0.087; TSS: mean ± SD = 0.807 ± 0.162). As predictor variables we included both annual climate variables (total annual precipitation, annual mean temperature) as well as seasonality variables (precipitation seasonality, precipitation of the driest quarter, temperature of the coldest month), derived from the Worldclim data set^61^. Given the choice of study area (i.e. continental Africa) we additionally included spatial filters to avoid over-prediction. The spatial filters are eigenvectors from a principal coordinate analysis of geographic coordinates^62^ which allow for inclusion of spatially-structured constraints (e.g. dispersal limitation) that go beyond the effects of included environmental predictor variables^63^. All continuous suitability surfaces of SDMs were later translated into binary species distribution maps using thresholds based on the best receiver operating characteristic curves (ROC) and True Skill Statistics (Supplementary Material)^64^. All modelled distributions were finally cross-checked and validated by a taxonomic expert (P. Wagner). The number of predictor variables was adjusted to improve the prediction of species distributions in case the expert-based knowledge suggested overfitting or overprediction (Supplementary Material). For a few species (*n* = 4), we used consensus SDMs (i.e. including areas for which two different models predicted a presence) because TSS and ROC provided undistinguishable distribution maps. For a few other species (*n* = 7), the shortage of locality records did not allow to accurately predict their known distributional range. In this case, we included expert-based range maps with the predicted distributions. Overall, the species distribution maps can be considered as model-assisted range maps. A detailed description of included predictor variables, SDM algorithms, model statistics, and selection of final maps is provided in Supplementary Material. The digital distribution maps and the occurrence records are available at the Dryad Repository (dx.doi.org/10.5061/dryad.kf154).

From the species distributions, we constructed a species richness map (Fig. 1c) by overlaying the estimated distributions of all 74 Agaminae species onto a grid in cylindrical equal area projection with *c.* 110 × 110 km resolution (equivalent to c. 1° × 1° near the equator). We used the observed localities for species with <5 records (*n* = 17 species) and the modelled distributions for the other species (*n* = 57 species) as described above.

### Historical colonization and dispersal

We used the recently developed software KISSMig^11^ — a simple, raster-based, stochastic dispersal model — to generate maps of geographic accessibility from areas of origin. In contrast to other migration models which require species-specific values of dispersal ability or demographic parameters, KISSMig requires no a priori knowledge about dispersal parameters^11^. As input, the software only needs a raster map, a suitability value for each raster cell, a defined point or area of origin, and the number of iterations of the simulation. Starting from the point of origin, KISSMig then iteratively uses a 3 × 3 cell algorithm to simulate spatial spread patterns on top of the suitability map. While such simulated spread patterns are necessarily simplistic, they do allow to uncover the broad-scale influence of limited migration on species distributions and species richness. KISSMig therefore allows to generate and test hypotheses about the relative influence of spread patterns on the spatial distribution of biodiversity^11^.

For the simulations, we identified the Sinai and the Bab al-Mandab (the 27 km broad strait between the Arabian Peninsula and the African continent) as the origins of colonization of agamid lizards into Africa (Fig. 2). This was based on phylogenetic, morphological and fossil evidence^28^,^29^,^31^,^32^. For the entire subfamily, we then simulated the subsequent dispersal into Africa from these areas of origin using four simple dispersal scenarios (DISP1–4, see details in Table 1). These four scenarios mainly differed in the way how dispersal suitability was quantified and hence produced different maps of spatial spread patterns into the continent (Fig. 2b–e). DISP1 assumed no barriers and equal suitability across Africa (a ‘null pattern’); DISP2 assumed zero suitability of the Sahara desert (a major dispersal barrier at least for 7 mya^18^); DISP3 assumed the same as DISP2 and in addition the Congo forests with low suitability (the central location of these rainforests has been similar over time^38^,^39^, and agamid lizards seem to have never colonized these rainforest habitats in Africa); and DISP4 allocated the highest suitability to arid corridors (hypothesized to be major dispersal routes^24^,^25^,^26^) and intermediate suitability to all other grid cells, except those encompassing the Sahara and Congo forests (Fig. 2a). The latter ones were given low suitability as in DISP3. For all four scenarios we used the minimum number of iterations to allow dispersal across all Africa (*n* = 700 for DISP1–3; *n* = 1000 iterations for DISP4). All simulations were done on a high-resolution 10 × 10 km grid and later averaged in ArcGIS (version 10.2, ESRI, Redlands, CA, USA) with the Spatial Analyst (mean in Zonal Statistics) at the same resolution (*c.* 110 × 110 km) as the species richness data (Fig. 2b–e).

### Other predictor variables to explain species richness

In addition to the simulated dispersal routes, we used other predictor variables related to current climate (six variables), past climate (six variables), and topographic heterogeneity (one variable) to explain species richness of agamid lizards across Africa (Table 1).

To quantify the effect of current climate we included six variables from the Worldclim dataset (www.worldclim.org)^61^ (Table 1). We chose annual mean temperature (TEMP) and annual precipitation (PREC) as well as temperature extremes (TEMP MAX, TEMP MIN), extremes of drought (PREC DRY), and precipitation seasonality (PREC SEAS). Other climate variables (e.g. temperature seasonality, precipitation of wettest month, etc.) were highly correlated with these climatic predictors (Spearman rank correlations *r* > 0.70) and hence not included. All climate data were assembled in ArcGIS and averaged from the original resolution of *c*. 1 km^2^ to the resolution of the species richness data (*c.* 110 × 110 km).

In addition to current climate, we also assembled six paleoclimatic predictor variables (Table 1). We used recently published paleoclimatic reconstructions from coupled ocean–atmosphere general circulation models for the Last Glacial Maximum (LGM) in the Late Pleistocene *c.* 21,000 years before present^65^, for the late Pliocene *c.* 3.29–2.97 mya^66^, and for the late Miocene *c.* 11.61–7.25 mya^39^. For each epoch, we calculated climate anomalies, i.e. the difference between current and past climate (i.e. contemporary climate minus paleoclimate), separately for annual mean temperature and annual precipitation, respectively (Table 1). Representing paleoclimate by anomalies is conservative, as only patterns related to deviations from current climate relationships will be ascribed to paleoclimate^6^,^7^. For the LGM, we used two different climate simulations (the Community Climate System Model version 3, CCSM3, and the Model for Interdisciplinary Research on Climate version 3, MIROC3.2) from the Paleoclimate Modeling Intercomparison Project PIMP2^65^. We then computed the mean values across both climate simulations. All palaeoclimate layers were resampled with a bilinear interpolation to the same resolution (30 arc seconds) as the present-day climate data^61^ before calculating the means per 110 × 110 km grid cell. Overall, this represented precipitation and temperature anomalies for the LGM (LGM TEMP, LGM PREC), the Pliocene (PLIO TEMP, PLIO PREC), and the Miocene (MIO TEMP, MIO PREC). Large positive anomaly values indicate a higher precipitation and temperature in the present than in the past, whereas small or negative anomaly values indicate the opposite, i.e. higher precipitation and temperature in the past than in the present.

We quantified heterogeneity in topographic relief for each 110 × 110 km grid cell as elevational range (maximum minus minimum elevation) from the 30″ resolution Shuttle Radar Topography Mission (SRTM) dataset^67^, downloaded from the Worldclim website (www.worldclim.org).

### Statistical analysis

We first tested which of the four dispersal scenarios (DISP1–DISP4) could best explain the current distribution of agamid species richness across Africa. For this, we calculated Spearman rank correlations (*r*) between species richness and all four dispersal route scenarios (DISP1–DISP4), respectively. We further partitioned the variation of species richness into components accounted for by the four dispersal scenarios and their combined effects^68^. We then used the best dispersal route scenarios (with the highest *r* and *R*^2^) as predictor variables in subsequent multi-predictor models to test their importance in explaining species richness relative to other predictor variables (see below). We tested the correlations with datasets that either included or excluded grid cells with species richness = 0 (compare Fig. 1c). Both approaches yielded qualitatively similar results. We only present the results for including all grid cells.

In a second step, we built ordinary least squares (OLS) multi-predictor regression models to test the relative importance of predictor variables in explaining species richness of agamid lizards across Africa. We first examined collinearity among all predictor variables. The variable PLIO PREC showed a high correlation with current PREC (Spearman rank *r* = −0.85), and current TEMP had a high variance inflation factor (VIF > 33). Both variables (PLIO PREC, TEMP) were therefore removed from the multi-predictor model. All other predictor variables showed low to intermediate collinearity (*r* < 0.62 and VIF < 5) and were hence included. The multi-predictor model with the included predictor variables was then subjected to a stepwise, backward model selection based on the Akaike Information Criterion (AIC) to select the most parsimonious multivariate model by minimizing AIC^33^. Model residuals were approximately normally distributed. Partial residual plots were used to identify heteroscedasticity and non-linearity. As a consequence, two variables (PREC DRY, and absolute values of LGM PREC) were log(x + 1) transformed; the remaining variables remained untransformed. A second order polynomial term was included for PREC SEAS. All predictor variables were centred to make effect sizes comparable (standardized coefficients). We included grid cells with >50% land and with species richness ≥0 (*n* = 2382), but qualitatively similar results were obtained when using only grid cells with species richness ≥1 (*n* = 1715). We tested multi-predictor regression models with either dispersal scenario DISP3 or DISP4 to represent the effect of historical dispersal limitation. Both yielded qualitatively similar results, though the effect of dispersal limitation was less pronounced in the model with DISP3 compared to the model with DISP4 (see Supplementary Material).

In a third step, we tested to what extent spatial autocorrelation might affect the results. The presence of spatial autocorrelation is problematic for classical statistical tests due to the inflation of type I errors which could affect the significance and interpretation of regression coefficients^68^. We therefore used Moran’s *I* values (calculated with the eight nearest neighbours) to test for the presence of spatial autocorrelation in the residuals of the multi-predictor OLS model. Because Moran’s *I* values were significant for OLS model residuals, we implemented a spatial simultaneous autoregressive (SAR) model to account for residual spatial autocorrelation^34^. SAR models supplement OLS regression with a spatial weights matrix that accounts for spatial autocorrelation in model residuals. We used a SAR model of the error type with a row-standardization for the spatial weights matrix as suggested by Kissling and Carl^34^. We defined the neighbourhood of the spatial weight matrix with the median distance to connect a grid cell to the eight nearest neighbours (157 km). For the SAR models, we quantified the explained variance of the environmental predictors (*R*^2^_*PRED*_) and the total explained variance (*R*^2^_*FULL*_) of the full SAR models (including environmental predictors and the spatial weights matrix)^34^. This was done using pseudo-*R*^2^ values, calculated as the squared Pearson correlation between predicted and true values^34^. We present results from both OLS and SAR models because potential shifts in model coefficients between non-spatial and spatial regression models might affect our ability to evaluate the importance of explanatory variables^69^. We consider variables that are statistically significant predictors in both OLS and SAR models as key predictors.

All statistical analyses were done in R version 3.2.2^70^. Variance partitioning was performed using the R package ‘vegan’ version 2.3–5, spatial analysis using the R package ‘spdep’ version 0.5–88, and dispersal route simulations using the R package ‘kissmig’ version 1.0–3^11^.

## Additional Information

**How to cite this article**: Kissling, W. D. *et al*. Historical colonization and dispersal limitation supplement climate and topography in shaping species richness of African lizards (Reptilia: Agaminae). *Sci. Rep.* **6**, 34014; doi: 10.1038/srep34014 (2016).

## Supplementary Material

Supplementary Information

## Figures and Tables

**Table 1:** Predictor variables to explain spatial variation in species richness of agamid lizards across Africa.

Abbreviation	Predictor variables (units)	Data source
*Simulated dispersal (accessibility)*		
DISP1	Simulated dispersal into a homogeneous environment (all grid cells with suitability *s* = 1), i.e. accessibility (simulated number of first occurrences) from colonization areas at Sinai and Bab al-Mandab (compare Fig. 2)	Locations of colonization areas from Wagner^26^, simulation of accessibility with KISSMig^11^
DISP2	Simulated dispersal (i.e. accessibility) as in DISP1, but Sahara desert masked as unsuitable (*s* = 0)	Extent of Sahara desert biome from Olson *et al*.^12^, origin of colonization from Wagner^26^, simulation with KISSMig^11^
DISP3	Simulated dispersal (i.e. accessibility) as in DISP2, but Congo forests with low suitability (*s* = 0.2)	Extent of Sahara desert and Congo rainforest from Olson *et al*.^12^, origin of colonization from Wagner^26^, simulation with KISSMig^11^
DISP4	Simulated dispersal (i.e. accessibility) as in DISP3, but arid corridors with high suitability (*s* = 1) and other cells with intermediate suitability (*s* = 0.5), except Sahara desert (*s* = 0) and Congo forests (*s* = 0.2)	Extent of Sahara desert and Congo rainforest from Olson *et al*.^12^, arid corridors and origin of colonization from Wagner^26^, simulation with KISSMig^11^
*Current climate*		
TEMP	Annual mean temperature (°C × 10)	Worldclim dataset^61^
TEMP MAX	Maximum temperature of the warmest month (°C × 10)	Worldclim dataset^61^
TEMP MIN	Minimum temperature of the coldest month (°C × 10)	Worldclim dataset^61^
PREC	Annual precipitation (mm yr^−1^)	Worldclim dataset^61^
PREC DRY	Precipitation of driest month (mm)	Worldclim dataset^61^
PREC SEAS	Precipitation seasonality: coefficient of variation of monthly values (mm)	Worldclim dataset^61^
*Paleoclimate*		
LGM TEMP	Anomaly in annual mean temperature between Last Glacial Maximum (*c.* 21,000 years ago) and present (°C × 10)	Calculated in ArcGIS as the difference between current annual mean temperature^61^ and annual mean temperature during the Last Glacial Maximum, the latter as a mean of the CCSM3 and MIROC3.2 models from the PIMP 2 project^65^, downloaded from Worldclim
LGM PREC	Anomaly in annual precipitation between Last Glacial Maximum (*c.* 21,000 years ago) and present (mm yr^−1^)	Calculated in ArcGIS as the difference between current annual precipitation^61^ and annual precipitation during the Last Glacial Maximum, the latter as a mean of the CCSM3 and MIROC3.2 models from the PIMP 2 project^65^, downloaded from Worldclim
PLIO TEMP	Anomaly in annual mean temperature between late Pliocene (*c.* 3 mya) and present (°C × 10)	Calculated in ArcGIS as the difference between current annual mean temperature^61^ and annual mean temperature during the late Pliocene^66^, the latter resampled in ArcGIS with a bilinear interpolation to the resolution of the current data
PLIO PREC	Anomaly in annual precipitation between between late Pliocene (*c.* 3 mya) and present (mm yr^−1^)	Calculated in ArcGIS as the difference between current annual precipitation^61^ annual precipitation during the late Pliocene^66^, the latter resampled in ArcGIS with a bilinear interpolation to the resolution of the current data
MIO TEMP	Anomaly in annual mean temperature between late Miocene (11.61–7.25 mya) and present (°C × 10)	Calculated in ArcGIS as the difference between current annual mean temperature^61^ and annual mean temperature during the late Miocene^39^, the latter resampled in ArcGIS with a bilinear interpolation to the resolution of the current data
MIO PREC	Anomaly in annual precipitation between late Miocene (11.61–7.25 mya) and present (mm yr^−1^)	Calculated in ArcGIS as the difference between current annual precipitation^61^ and annual precipitation during the late Miocene^39^, the latter resampled in ArcGIS with a bilinear interpolation to the resolution of the current data
*Topographic heterogeneity*		
TOPO	Topographic heterogeneity: range in elevation (m)	SRTM data^67^ downloaded from Worldclim and processed in ArcGIS

**Table 2:** Standardized coefficients from multi-predictor regression models to explain species richness of agamid lizards across Africa.

	OLS		SAR	
	Coefficient	*p*	Coefficient	*p*
Intercept	1.885	***	1.819	***
DISP4	**0.542**	***	**0.306**	***
TEMP MAX	**−0.146**	**	**−0.210**	*
TEMP MIN	0.333	***	**−**0.126	n.s.
PREC	**−**0.251	***	**−**0.152	n.s.
PREC DRY	**−**0.107	*	**−**0.021	n.s.
PREC SEAS	**−0.320**	***	**−0.313**	***
PREC SEAS^2^	**−**0.373	***	0.144	**
LGM TEMP	**0.096**	**	**0.368**	***
LGM PREC	0.350	***	0.011	n.s.
PLIO TEMP	**−**0.296	***	**−**0.102	n.s.
MIO TEMP	**0.314**	***	**0.172**	*
MIO PREC	0.067	n.s.	0.022	n.s.
TOPO	**0.546**	***	**0.217**	***
*R*^*2*^_*PRED*_	0.450		0.276	
*R*^*2*^_*FULL*_	**—**		0.891	
Moran’s *I*	0.721		**−**0.012	
*p* of Moran’s *I*	***		n.s.	

Two types of models are compared, a non-spatial ordinary least square (OLS) regression and a spatial simultaneous autoregressive (SAR) model. Significant linear effects detected in both OLS and SAR models are indicated by boldface type. PREC DRY and absolute values of LGM PREC were log(x + 1) transformed, all other predictor variables and the response variable (species richness) were untransformed (compare Table 1 for abbreviations and explanations of predictor variables). The explained variance of the environmental components (*R*^2^_*PRED*_), the explained variance of the full SAR model including both environment and space (*R*^2^_*FULL*_), the Moran’s *I*, and the *p*-value of Moran’s *I* are given. Significance of Moran’s *I* was determined by permutation tests (*n* = 999 permutations). Significance levels: ^***^p < 0.001; ^**^p < 0.01; ^*^p < 0.05. n.s., not significant.
