# Supplementary material for: Historical colonization and dispersal limitation supplement climate and topography in shaping species richness of African lizards (Reptilia: Agaminae)
Source: Sci Rep. 2016 Sep 27;6:34014. doi: 10.1038/srep34014 (PMC5037428; doi:10.1038/srep34014)
Supplement: Supplementary Information [file srep34014-s1.doc]

**SUPPLEMENTARY MATERIAL**

Historical colonization and dispersal limitation supplement climate and topography in shaping species richness of African lizards (Reptilia: Agaminae)

By W. Daniel Kissling, Anne Blach-Overgaard, Roelof E. Zwaan & Philipp Wagner

**Additional information on species occurrences, taxonomy, species distribution modelling and species richness analyses**

**Database of locality records**

Sources of information

We compiled the most comprehensive geographic database to date of occurrence records for the 74 African species in the reptile subfamily Agaminae (genera *Acanthocercus*, *Agama*, *Pseudotrapelus*, *Trapelus* and *Xenagama*). A total of 16,802 available occurrence records were extracted from the following sources:

(1) Collections linked to the Global Biodiversity Information Facility (GBIF, [http://data.gbif.org](http://data.gbif.org/)).

(2) The HerpNET database (<http://www.herpnet.org/>).

(3) Additional museum collections. This included directly accessed specimen (i.e. handheld inspections by P. Wagner) from the following museums: National Museums of Kenya (NMK), Nairobi, Kenya; American Museum of Natural History (AMNH), New York, USA; Natural History Museum (BMNH), London, UK; California Academy of Sciences (CAS), San Francisco, USA; Museo Civico di Storia Naturale - Giacomo Doria (MCSN), Genova, Italy; Museum of Comparative Zoology (MCZ), Harvard, USA; Muséum d’histoire naturelle de la ville de Genève (MHNG), Genève, Switzland; Muséum nationale d’histoire naturelle (MNHN), Paris, France; Museum of Vertebrate Zoology (MVZ), Berkeley, U.S.A.; Museo di Storia Naturale “La Specola” (MZUF), Florence, Italy; Naturhistorisches Museum (NHMW), Wien, Austria; Senckenberg Museum (SMF), Frankfurt, Germany; Zoologisches Forschungsmuseum Alexander Koenig (ZFMK), Bonn, Germany; Museum für Naturkunde (ZMB), Berlin, Germany; Zoologische Staatssammlung München (ZSM), München, Germany.

(4) Complementary literature

(5) Private databases and observations held by two field herpetologists (A.M. Bauer and P. Wagner).

Most of these occurrences (i.e. 11,597 records, 69%) were either not georeferenced (i.e. only locality names or country-level information available, but no latitude-longitude coordinates) or contained doubtful taxonomic or wrong locality information (see below section on quality checking). Hence, we only included records for which latitude-longitude information within Africa was available and for which taxonomic and locality information was considered to be reliable. Georeferenced locality records of introduced species were excluded as well as records pre-dating 1950. This left a total of 5,205 georeferenced and quality-checked occurrence records across all Agaminae species (Table S1). Of those records, 3,453 (66%) were geographical duplicates (i.e. with identical latitude-longitude coordinates for the same species coming from different sources). This left 1,752 (34%) records with geographically unique georeferences (Table S1). For the species distribution modelling performed at 10 × 10 km resolution (see below), we used 1,454 records as only those records were geographically unique at this spatial grain size. Thus, from the original 16,802 available occurrence records 1,454 (9%) were finally considered to be useful (i.e. georeferenced), reliable (geographically and taxonomically checked), and geographically unique (at the 10 × 10 km resolution). We specifically searched for additional georeferenced locality records (from sources 3–5 above) for parts of a species geographic range that were not satisfactorily covered by the standard online data sources (GBIF and HerpNET, sources 1–2 above). This ensured a relatively good coverage of georeferenced and quality-checked locality records across the known geographic ranges of most species. For a few species (*n* = 7, 9%), the collected locality records did not satisfactorily cover the whole known geographic range of a species and additional expert knowledge had to be used to estimate the known distribution of those species (see below ‘Species distribution modelling’). The total number of locality records varied among species (median = 13; mean ± SD = 20 ± 20) and the full overview is provided in Table S1.

Quality checking of data

Each of the 16,802 available occurrence records was meticulously scrutinized for any geographical or taxonomic issue. This included the correction (or exclusion) of faulty georeferences and taxonomically doubtful taxa names. To identify geographical biases in the locality records, we plotted all georeferenced occurrence records for each individual species separately and identified distributional outliers that clearly fall outside the known distribution. We further plotted all georeferenced occurrence records for each country to identify outliers (note that country information is specified as additional information in the original data tables, independent from latitude-longitudes, and therefore can be used for identifying outliers). Faulty georeferences were corrected whenever possible and necessary. This included wrong signs (e.g. missing minus signs) in front of latitude or longitude coordinates (in decimal degrees) in the original data sources as well as georeferenced locality records that fell outside the countries in which they were supposed to occur. Latitude-longitude coordinates that fell into the sea, outside the African continent, or into grid cells without environmental data (see below ‘Predictor variables for modelling individual species distributions’) were also excluded or corrected were possible. In addition to identifying geographic biases in our data, we also performed a major effort in quality checking of taxonomic names. Wrong species identifications were rectified, old names (e.g. former subspecies now considered as species) were adjusted, and all doubtful species identifications were excluded (see below section ‘Taxonomy’ for more details).

**Taxonomy**

To harmonize the different datasets, a number of taxonomic changes and corrections at subspecies, species and genera level had to be applied to the taxon names retrieved from the databases of locality records (see above). This was done by our taxonomic expert of this subfamily (P. Wagner). If not yet published, the usage of these names and changes is provisional, but it guarantees a consistent terminology across these heterogeneous collection names. The species list below includes agamid taxa of the subfamily Agaminae (genera *Acanthocercus*, *Agama*, *Pseudotrapelus*, *Trapelus*, and *Xenagama*). The subfamily Uromastycinae (genus *Uromastyx*) is not included. Our work considers a total of 74 recognized species, including 13 *Acanthocercus*, 50 *Agama*, 1 *Pseudotrapelus*, 6 *Trapelus*, and 4 *Xenagama* species (Table S1). In the following, we describe the applied taxonomy within these five genera of Agaminae.

*Acanthocercus*

We considered a total of 13 taxa within the genus *Acanthocercus* (Table S1). Species within the genus *Acanthocercus* Fitzinger, 1843 predominantly occur in the eastern parts of Africa. This genus is currently in need of a taxonomic revision (P. Wagner, own data). We recognize the six currently known African species: *A. annectans*, *A. atricollis* (including several subspecies and the closely related *A. cyanocephalus*), *A. branchi*, *A. cyanogaster*, *A. guentherpetersi*, and *A. phillipsi*. The former species *Acanthocercus* *zonurus* is now recognized in the genus *Xenagama* . In addition to the six species, we also recognize the known subspecies of *A. atricollis* as distinct taxa: *A. gregorii*, *A. kiwuensis*, *A. loveridgei*, *A. minutus*, and *A. ugandaensis*. Furthermore, *A. cyanocephalus* sometimes recognized as synonym of *A. atricollis* and a so far undescribed form *Acanthocercus* aff. *atricollis* are also recognized by us separately.

*Agama*

The genus *Agama* Daudin, 1802 is by far the most species-rich genus in the subfamily Agaminae and its species occur across most parts of Africa. We distinguish a total of 50 species (Table S1). We follow the definition of *Agama agama* as a monotypical species of northern Central Africa . Therefore, we recognize *Agama wagneri* as a synonym of *A. agama*. For populations in West and southern Central Africa the name *Agama picticauda* Peters, 1877 is available and the former subspecies or synonyms, *Agama agama africana*, *Agama agama musocosensis*, and *Agama congica*, were here recognized as full species . *Agama lionotus*, formerly also known as subspecies of *A. agama*, was recognized as an own species , while *A. agama usambarae* was treated as a synonym of *A. lionotus* . Anticipating further research (P. Wagner et al., own data), we recognize *A. dodomae* at the species level, and *A. lionotus elgonis* as well as *A. dodomae ufipae* at the subspecies level.

Several taxa currently known as subspecies have to be recognized as full species or synonyms. The former subspecies *Agama aculeata distanti*, *A. doriae benueensis*, and *A. planiceps schacki* are recognized at species rank, while *Agama castroviejoi* was recognized as synonym of *Agama boueti*. We also recognize taxa such as *Agama bottegi* and *Agama hartmanni* at the species level. We acknowledge the species *Agama robecchii* as a non-*Agama* species of unknown generic assignment, but given current lack of knowledge we still use the species name *Agama robecchii* here.

Some very recently described cryptic species, such as *Agama hulbertorum* , were not included in the species distribution modelling because such species were described after the analysis and data compilation.

*Pseudotrapelus*

For the genus *Pseudotrapelus* Fitzinger, 1843 we included one species (*Pseudotrapelus sinaitus*) (Table S1). This species represents most probably a species complex . However, no further information is currently available and we therefore treat this taxon here as one species.

*Trapelus*

The genus *Trapelus* Cuvier, 1817 is recognized here with six species (Table S1). Four of those species (*T. boehmei*, *T. mutabilis*, *T. schmitzi* and *T. tournevillei*) have been described for Northern Africa and one (*T. savignii*) reaches the African continent in Egypt. *Trapelus asperses*, currently recognized as synonym of *T. mutabilis*, is recognized here as a valid species anticipating a major revision of the African *Trapelus* taxa . *Trapelus pallidus* is here included as a subspecies of *T. mutabilis*.

*Xenagama*

We recognize four *Xenagama* species (Table S1). Species in the genus *Xenagama* Boulenger, 1895 are restricted to the Horn of Africa. The genus was very recently reviewed and includes the species *X. batillifera*, *X. taylori*, *X. wilmsi* and *X. zonura*. The latter taxon was formerly recognized in the genus *Acanthocercus* (see above).

**Predictor variables for modelling individual species distributions**

We initially selected six bioclimatic variables as potential predictors for modelling species distributions. We chose two annual climate variables (total annual precipitation and annual mean temperature) as well as four seasonality predictors (precipitation seasonality measured as coefficient of variation of monthly values, precipitation of the driest quarter, temperature of the coldest month, and temperature seasonality measured as standard deviation * 100 of monthly mean values). We selected these complementary predictor variables because both annual and seasonality variables of temperature and precipitation are thought to be the strongest determinants of broad-scale distributions of ectotherms such as reptiles . Temperature seasonality was later excluded because it highly correlates with precipitation and temperature of the coldest month (Spearman’s rank correlations *r* ≥ 0.7). Hence, a maximum of five climate variables was used in the species distribution modelling (see below). Climate data were derived from the Worldclim data set ([www.worldclim.org](http://www.worldclim.org/)) which represents interpolated climate surfaces for global land areas based on climate records from the 1950–2000 period provided at a 5 arc-minute grid resolution. The climatic data was subsequently projected to the Lambert Azimuthal Equal area projection and resampled with a bilinear interpolation to a 10 × 10 km resolution before further geoprocessing.

In addition to climate data, we accounted for potential broad-scale spatial constraints on species distributions using spatial filters . These spatial filters reduce over-prediction and improve model performance in areas were spatially-structured constraints (e.g. dispersal limitation) beyond the effects of included environmental variables limit the distribution of a particular species . Spatial filters were computed as eigenvectors derived from a principal coordinate analysis based on a pairwise distance matrix of geographical coordinates (latitude and longitude) of centroids of all grid cells across the study area. Hence, spatial filters are orthogonal variables which represent the spatial relationship amongst sampling units (here grid cells) at various scales from broad- to fine-scale spatial patterns. The spatial filters were computed in SAM 3.0 in a Lambert Azimuthal Equal area projection using default settings. Spatial filters were further geoprocessed in ArcGIS as described in a previous paper and then resampled to 10 × 10 km resolution similar to the climatic data (see above). All GIS operations were conducted in ArcGIS 9.3 (ESRI, Redlands, CA, USA).

**Species distribution modelling**

Model types

Due to differences among species in the number of unique locality records at 10 × 10 km resolution (Table S1), we estimated the distributions of Agaminae species across continental Africa in a three-way approach. For species with <5 records (*n* = 17 species, Table S1), we used the observed records rather than modelled distributions because sample size was too low for a meaningful implementation of models. For species with ≥5 unique locality records at 10 × 10 km resolution, we employed species distribution models (SDMs) using two different ways depending on sample size of locality records. For species with sample sizes 20 > *x* ≥ 5 (*n* = 31 species, Table S1), we used a simple bioclimatic envelop model referred to as surface range envelope (SRE) in the R package ‘biomod2’ version 2.1.9 . Note that this SRE model is similar to a BIOCLIM model . For species with ≥20 records (*n* = 26 species, Table S1), we used SRE, but also advanced SDMs based on machine-learning methods such as Maxent (MAX) and generalized boosting models (GBM) . The latter two methods are also implemented in the R package ‘biomod2’ version 2.1.9 (Thuiller et al. 2009). Note that GBM is alternatively known as Boosting Regression Tree . For MAX, we chose the default settings of ‘biomod2’ but set the maximum iterations to 500 to make all default settings similar to the desktop version of MAX . These settings have been shown to provide overall robust results . For GBM, we chose the default settings of ‘biomod2’ running a maximum of 500 trees and five cross validations to select the optimal number of trees. For both MAX and GBM, the continuous suitability surfaces had to be translated into binary predictions to make a species distribution map. We used two different thresholds that were found to produce the best predictions : one which maximises the sum of sensitivity and specificity of a given model based on the receiver operating characteristic curve (ROC), and one that maximises the true skill statistic TSS (i.e., the sum of sensitivity and specificity minus one). SRE models do not need such thresholds because their predictions are already binary. Hence, in all SRE models we chose to use the complete predicted range (0-100%). All species distribution modelling was done using the software R 2.15.2 .

Model statistics for species with ≥20 records

For both MAX and GBM, we used 10,000 randomly selected pseudo-absences from across the study area of continental Africa. For each species, the presence data was randomly divided in calibration (80%) and test (20%) datasets for model evaluation. This procedure was repeated 10 times for each algorithm, but final models were fitted on 100% of the occurrence data. To assess the performance of all SDM algorithms (including MAX, GBM and SRE), we used both the threshold-independent measure area under the receiver operating characteristic curve (AUC) and the threshold-dependent TSS . Both AUC and TSS were only provided for species ≥20 records because a low sample size makes AUC and TSS scores highly variable among the 10 replicated models and hence random test samples can easily bias these test scores (e.g. by selecting a test record that is climatically very distant from the calibration sample). Models with AUC ≥ 0.750 and TSS ≥ 0.400 are generally considered to be good and reliable predictions. The finally selected models (see below and Table S1) all showed good predictions according to AUC (mean ± SD = 0.905 ± 0.087) and TSS (mean ± SD = 0.807 ± 0.162).

Selection of final distribution maps

The use of large spatial extents for selecting background data for narrowly-distributed species has been questioned in previous comparative studies because model test statistics (e.g. AUC, TSS) might be inflated and models can potentially overpredict species distributions (e.g., Elith *et al.* 2011, Merow *et al.* 2014). We accounted for this by 1) including spatial filters as predictor variables to remove overprediction at a continental scale (see above), and 2) selecting the most realistic distribution maps for each species independent of model test statistics. The latter was done using an iterative approach of model predictions and expert knowledge to select the final distribution maps of all species. The aim was to find the best suitable combination of algorithm, predictors and threshold statistics guided and combined with expert knowledge. Initial models (MAX, GBM and SRE) were run with the five climate predictors and the first five broad-scale spatial filters (see above). MAX and GBM models were then translated into binary predictions using ROC and TSS thresholds (see above). Each binary species distribution map was visually inspected by an expert of this reptile subfamily (P. Wagner). The final distribution map was selected by the expert in case the model had predicted the known distribution well. In a number of cases, however, the initial models did not predict spatial species distributions well, e.g. due to overfitting or overprediction of a species’ distribution. For those species, the models were improved by adjusting the number of predictors (both climate and spatial filters). This was done by 1) reducing the number of climate predictors to three seasonality variables or to the two annual predictors, respectively, and combining those with the same number of spatial filters (for species distributions which were overfitted), or 2) including more (up to seven) spatial filters (for species distributions which were clearly overpredicted). The best distribution map was than selected by the expert among all available predictions. For 4 species, MAX predictions provided undistinguishable distribution maps for the two thresholds (TSS or ROC). In these cases, we used a consensus of the two models, extracting those areas for which both models predicted a presence (Table S1). Altogether, the initial and improved models provided satisfactory range maps for 66 out of 74 species (89%). Only 8 species (11%) were left with predicted species distributions that were incongruent with expert knowledge. One of those species (*Agama anchietae*) occurs only in south-western Africa, but models predicted the presence of the species along the coast of North Africa. This clearly wrong prediction was therefore manually removed in GIS (see Supporting Information Appendix 2). For the remaining species (*n* = 7), we were unable to satisfactorily predict their geographic distribution by modelling. This was most likely due to a shortage of locality records in some parts of their distributional range. To provide a final distribution map for those species (compare Table S1), we therefore added distributional areas by hand onto the modelled distributions based on the best expert knowledge currently available (see available data at LINK TO BE ADDED ONCE PAPER IS ACCEPTED). The final species distribution maps can therefore be considered as model-assisted range maps.

**Species richness analyses**

We implemented multi-predictor regression models to explain species richness of agamid lizards across Africa (see Methods in main text for details). Since two simulated dispersal scenarios (DISP3 and DISP4, compare Table 1 in main text) were almost equally relevant to explain current species richness in univariate analyses (see results in main text), we implemented multi-predictor regression models with either dispersal scenario DISP3 or DISP4 to test for the effect of historical dispersal limitation on species richness (Table S2). The effect of DISP3 (standardized coefficient: 0.331) was less pronounced then DISP4 (standardized coefficient: 0.542), consistent with the smaller Spearman rank correlation in the univariate analyses (DISP3: *r* = 0.42; DISP4: *r* = 0.45; see results in main text). Otherwise, the relative effects of climatic and topographic variables were qualitatively similar between both models (Table S2). We only use the multi-predictor regression model with DISP4 in the main manuscript.

**References**

**Table S1**: Overview of occurrence records and species distribution modelling. Total records include the sum of duplicated records and unique records. The number of records for modelling is smaller than the number of unique records because the spatial resolution for modelling is 10 × 10 km. For species with ≥5 unique locality records at 10 × 10 km resolution, three different types of species distribution models were used (SRE = surface range envelope, MAX = Maxent, GBM = generalized boosting models). Species with <5 records were not modelled (model type = ‘None’). For MAX and GBM, two different threshold methods for translating the continuous suitability surfaces into binary predictions were used based on 1) the true skill statistic (TSS; where TSS is maximised), and 2) the receiver operating characteristic curve (ROC; maximising the sum of specificity and sensitivity). The number of predictors includes both climate variables as well as spatial filters (see text for details, NA = not available). Comments: 'Consensus model' = the two threshold approaches (TSS or ROC) provided undistinguishable distribution maps, hence a consensus of the two models was used (i.e. areas for which both models predicted a presence); 'Expert range map included' = distributional areas were added onto the modelled distributions based on expert knowledge; 'Overprediction removed' = clearly wrong prediction at the other end of the continent was removed manually.

|  |  |  |  |  |  |  |  |
| --- | --- | --- | --- | --- | --- | --- | --- |
| Taxon | Total records | Duplicated records | Unique records | Records for modelling | Model type | Number of predictors (climate + spatial filter) | Comment |
|  |  |  |  |  |  |  |  |
|  |  |  |  |  |  |  |  |
| Acanthocercus annectens | 21 | 2 | 19 | 13 | SRE | 5+6 |  |
| Acanthocercus atricollis | 50 | 8 | 42 | 35 | SRE | 5+6 |  |
| Acanthocercus aff. atricollis | 8 | 1 | 7 | 3 | None | NA |  |
| Acanthocercus branchi | 16 | 1 | 15 | 14 | SRE | 5+5 |  |
| Acanthocercus cyanocephalus | 40 | 5 | 35 | 26 | GBM (ROC) | 5+5 |  |
| Acanthocercus cyanogaster | 8 | 0 | 8 | 7 | SRE | 5+5 |  |
| Acanthocercus gregorii | 15 | 2 | 13 | 12 | SRE | 2+2 |  |
| Acanthocercus guentherpetersi | 7 | 0 | 7 | 7 | SRE | 5+5 |  |
| Acanthocercus kiwuensis | 25 | 0 | 25 | 20 | GBM (ROC) | 5+6 |  |
| Acanthocercus loveridgei | 159 | 60 | 99 | 81 | Max (ROC/TSS) | 5+6 | Consensus model |
| Acanthocercus minutus | 64 | 26 | 38 | 28 | Max (ROC) | 5+5 |  |
| Acanthocercus phillipsi | 7 | 1 | 6 | 6 | SRE | 5+5 | Expert range map included |
| Acanthocercus ugandaensis | 222 | 138 | 84 | 53 | SRE | 5+6 |  |
| Agama aculeata | 923 | 853 | 70 | 59 | Max (ROC/TSS) | 5+6 | Consensus model |
| Agama africana | 31 | 14 | 17 | 17 | SRE | 5+5 |  |
| Agama agama | 25 | 18 | 7 | 7 | SRE | 5+5 |  |
| Agama anchietae | 88 | 48 | 40 | 31 | Max (ROC) | 5+5 | Overprediction removed |
| Agama armata | 53 | 26 | 27 | 18 | SRE | 5+6 | Expert range map included |
| Agama atra | 110 | 85 | 25 | 18 | SRE | 3+3 | Expert range map included |
| Agama benueensis | 28 | 0 | 28 | 26 | Max (ROC/TSS) | 5+6 | Consensus model |
| Agama boensis | 20 | 0 | 20 | 20 | Max (ROC) | 5+6 |  |
| Agama bottegi | 3 | 0 | 3 | 3 | None | NA |  |
| Agama boueti | 63 | 5 | 58 | 56 | GBM (ROC) | 5+5 |  |
| Agama boulengeri | 51 | 2 | 49 | 45 | GBM (ROC) | 5+7 |  |
| Agama caudospinosa | 195 | 173 | 22 | 15 | SRE | 5+5 |  |
| Agama congica | 4 | 1 | 3 | 3 | None | NA |  |
| Agama cristata | 5 | 0 | 5 | 3 | None | NA |  |
| Agama distanti | 27 | 17 | 10 | 7 | SRE | 5+5 |  |
| Agama dodomae | 138 | 113 | 25 | 21 | SRE | 5+5 |  |
| Agama doriae | 33 | 8 | 25 | 23 | SRE | 5+5 |  |
| Agama etoshae | 1 | 0 | 1 | 1 | None | NA |  |
| Agama finchi | 100 | 52 | 48 | 44 | Max (TSS) | 5+6 |  |
| Agama gracilimembris | 34 | 0 | 34 | 33 | Max (ROC/TSS) | 5+5 | Consensus model |
| Agama hartmanni | 7 | 0 | 7 | 7 | SRE | 5+5 |  |
| Agama hispida | 6 | 3 | 3 | 3 | None | NA |  |
| Agama impalearis | 106 | 81 | 25 | 22 | GBM (ROC) | 5+5 |  |
| Agama insularis | 3 | 0 | 3 | 3 | None | NA |  |
| Agama kaimosae | 31 | 21 | 10 | 8 | SRE | 5+5 |  |
| Agama kirkii | 21 | 5 | 16 | 16 | SRE | 5+5 |  |
| Agama knobeli | 19 | 10 | 9 | 9 | SRE | 5+5 |  |
| Agama lanzai | 6 | 1 | 5 | 2 | None | NA |  |
| Agama lebretoni | 121 | 74 | 47 | 34 | SRE | 5+5 |  |
| Agama lionotus | 778 | 632 | 146 | 105 | GBM (ROC) | 5+5 |  |
| Agama lucyae | 10 | 8 | 2 | 1 | None | NA |  |
| Agama makarikarica | 1 | 0 | 1 | 1 | None | NA |  |
| Agama montana | 62 | 50 | 12 | 11 | SRE | 5+5 |  |
| Agama mossambica | 79 | 59 | 20 | 18 | SRE | 5+5 | Expert range map included |
| Agama mucosoensis | 6 | 0 | 6 | 5 | SRE | 5+5 |  |
| Agama mwanzae | 100 | 90 | 10 | 9 | SRE | 5+5 |  |
| Agama parafricana | 4 | 0 | 4 | 4 | None | NA |  |
| Agama paragama | 51 | 0 | 51 | 49 | Max (ROC) | 5+5 |  |
| Agama persimilis | 17 | 0 | 17 | 15 | SRE | 5+5 |  |
| Agama picticauda | 305 | 223 | 82 | 71 | SRE | 5+5 |  |
| Agama planiceps | 101 | 87 | 14 | 9 | SRE | 3+3 | Expert range map included |
| Agama robecchii | 3 | 0 | 3 | 3 | None | NA |  |
| Agama rueppelli | 157 | 93 | 64 | 41 | SRE | 5+5 |  |
| Agama sankaranica | 50 | 9 | 41 | 38 | SRE | 5+5 |  |
| Agama schacki | 10 | 6 | 4 | 4 | None | NA |  |
| Agama somalica | 43 | 21 | 22 | 13 | SRE | 5+5 |  |
| Agama spinosa | 270 | 250 | 20 | 16 | SRE | 5+5 |  |
| Agama tassiliensis | 23 | 3 | 20 | 20 | GBM (ROC) | 5+5 |  |
| Agama turuensis | 5 | 3 | 2 | 2 | None | NA |  |
| Agama weidholzi | 35 | 0 | 35 | 31 | GBM (ROC) | 5+6 |  |
| Pseudotrapelus sinaitus | 7 | 1 | 6 | 6 | SRE | 3+3 | Expert range map included |
| Trapelus aspersus | 14 | 4 | 10 | 10 | SRE | 5+5 |  |
| Trapelus boehmei | 89 | 38 | 51 | 51 | GBM (ROC) | 5+5 |  |
| Trapelus mutabilis | 43 | 22 | 21 | 18 | SRE | 5+5 |  |
| Trapelus savignii | 2 | 0 | 2 | 2 | None | NA |  |
| Trapelus schmitzi | 2 | 0 | 2 | 2 | None | NA |  |
| Trapelus tournevillei | 6 | 0 | 6 | 6 | SRE | 5+5 |  |
| Xenagama batillifera | 12 | 0 | 12 | 10 | SRE | 5+5 |  |
| Xenagama taylori | 13 | 0 | 13 | 11 | SRE | 5+5 |  |
| Xenagama wilmsi | 10 | 0 | 10 | 10 | SRE | 3+3 | Expert range map included |
| Xenagama zonura | 3 | 0 | 3 | 3 | None | NA |  |
|  |  |  |  |  |  |  |  |
| **Total** | **5205** | **3453** | **1752** | **1454** |  |  |  |
|  |  |  |  |  |  |  |  |

**Table S2:** Comparison of standardized coefficients from ordinary least squares (OLS) multi-predictor regression models with either dispersal scenario DISP3 (left column) or DISP4 (right column) to represent the effect of historical dispersal limitation. For model selection and implementation, see methods and Table 2 in main text. Significance levels: ****p* < 0.001; ***p* < 0.01; **p* < 0.05. n.s., not significant

|  |  |  |  |  |  |
| --- | --- | --- | --- | --- | --- |
|  | OLS with DISP3 | |  | OLS with DISP4 | |
|  |  |  |  |  |  |
|  |  |  |  |  |  |
|  | Coefficient | *p* |  | Coefficient | *p* |
|  |  |  |  |  |  |
|  |  |  |  |  |  |
| Intercept | 1.885 | *** |  | 1.885 | *** |
| DISP3 or DISP4 | 0.331 | *** |  | 0.542 | *** |
| TEMP MAX | -0.222 | *** |  | -0.146 | ** |
| TEMP MIN | 0.321 | *** |  | 0.333 | *** |
| PREC | -0.281 | *** |  | -0.251 | *** |
| PREC DRY | -0.133 | ** |  | -0.107 | * |
| PREC SEAS | -0.222 | *** |  | -0.320 | *** |
| PREC SEAS^2^ | -0.415 | *** |  | -0.373 | *** |
| LGM TEMP | 0.124 | *** |  | 0.096 | ** |
| LGM PREC | 0.391 | *** |  | 0.350 | *** |
| PLIO TEMP | -0.214 | *** |  | -0.296 | *** |
| MIO TEMP | 0.304 | *** |  | 0.314 | *** |
| MIO PREC | – |  |  | 0.067 | n.s. |
| TOPO | 0.582 | *** |  | 0.546 | *** |
|  |  |  |  |  |  |
| *R^2^* | 0.422 |  |  | 0.450 |  |
|  |  |  |  |  |  |
|  |  |  |  |  |  |
